# Global epidemiology of yaws: a systematic review

**DOI:** 10.1016/S2214-109X(15)00011-X

**Published:** 2015-05-19

**Authors:** Oriol Mitjà, Michael Marks, Diby J P Konan, Gilbert Ayelo, Camila Gonzalez-Beiras, Bernard Boua, Wendy Houinei, Yiragnima Kobara, Earnest N Tabah, Agana Nsiire, Damas Obvala, Fasiah Taleo, Rita Djupuri, Zhang Zaixing, Jürg Utzinger, Lasse S Vestergaard, Quique Bassat, Kingsley Asiedu

**Affiliations:** a Barcelona Institute for Global Health, Barcelona Centre for International Health Research, Hospital Clinic, University of Barcelona, Barcelona, Spain; b Lihir Medical Centre–International SOS, Newcrest Mining, Lihir Island, Papua New Guinea; c Department of Clinical Research, London School of Hygiene and Tropical Medicine, London, UK; d Hospital for Tropical Diseases, University College London Hospitals NHS Trust, London, UK; e Laboratoire Biostatistique et d'Informatique Médicale, Université Félix Houphouët-Boigny, Abidjan, Côte d'Ivoire; f Centre de Dépistage et de Traitement de l'Ulcère de Buruli d'Allada, Cotonou, Benin; g Programme National de Lutte contre les Maladies Tropicales Négligées, Ministère de la Santé Publique, de la Population et de la Lutte contre le SIDA, Bangui, Central African Republic; h Disease Control Branch, National Department of Health, Port Moresby, Papua New Guinea; i Programme National de Lutte contre l'Ulcère de Buruli et la Lèpre, Lomé, Togo; j National Leprosy, Buruli Ulcer, Yaws and Leishmaniasis Control Programme, Ministry of Public Health, Yaoundé, Cameroon; k Ghana Health Service, Public Health Division, Accra, Ghana; l Programme National de Lutte contre l'Ulcère de Buruli, Ministère de la Santé Publique, Brazzaville, Congo; m Neglected Tropical Diseases Program, Public Health Directorate, Health Department, Port Vila, Vanuatu; n National Leprosy and Yaws Programme, Ministry of Health, Jakarta, Indonesia; o World Health Organization Country Office, Honiara, Solomon Islands; p WHO Country Office, Manila, Philippines; q Department of Epidemiology and Public Health, Swiss Tropical and Public Health Institute, Basel, Switzerland; r World Health Organization Regional Office for the Western Pacific, Manila, Philippines; s Department of Control of Neglected Tropical Diseases, World Health Organization, Geneva, Switzerland

## Abstract

**Background:**

To achieve yaws eradication, the use of the new WHO strategy of initial mass treatment with azithromycin and surveillance twice a year needs to be extended everywhere the disease occurs. However, the geographic scope of the disease is unknown. We aimed to synthesise published and unpublished work to update the reported number of people with yaws at national and subnational levels and to estimate at-risk populations.

**Methods:**

We searched PubMed and WHO databases to identify published data for prevalence of active and latent yaws from Jan 1, 1990, to Dec 31, 2014. We also searched for ongoing or recently completed unpublished studies from the WHO yaws surveillance network. We estimated yaws prevalence (and 95% CIs). We collected yaws incidence data from official national surveillance programmes at the first administrative level from Jan 1, 2010, to Dec 31, 2013, and we used total population data at the second administrative level to estimate the size of at-risk populations.

**Findings:**

We identified 103 records, of which 23 published articles describing 27 studies and four unpublished studies met the inclusion criteria. Prevalence of active disease ranged from 0·31% to 14·54% in yaws-endemic areas, and prevalence of latent yaws ranged from 2·45% to 31·05%. During 2010–13, 256 343 yaws cases were reported to WHO from 13 endemic countries, all of which are low-income and middle-income countries. 215 308 (84%) of 256 343 cases reported to WHO were from three countries—Papua New Guinea, Solomon Islands, and Ghana. We estimated that, in 2012, over 89 million people were living in yaws-endemic districts.

**Interpretation:**

Papua New Guinea, Solomon Islands, and Ghana should be the focus of initial efforts at implementing the WHO yaws eradication strategy. Community-based mapping and active surveillance must accompany the implementation of yaws eradication activities.

**Funding:**

None.

## Introduction

Yaws is a neglected tropical disease caused by *Treponema pallidum* subspecies *pertenue*.^1^ This bacterium causes a chronic relapsing non-venereal treponematosis, characterised by highly contagious primary and secondary cutaneous lesions and non-contagious tertiary destructive lesions of the bones. The infection can become latent at any time, with only serological evidence of infection, and relapses can occur for up to 5–10 years. The ratio of clinically apparent to latent cases has been estimated to be as high as 1:6.^1^

In 2012, WHO launched a new initiative to eradicate yaws by 2020.^2^ Undertaking surveys and mapping the disease at a community level and immediately treating the entire endemic community with single-dose azithromycin^3^ is recommended.^2^ The efficacy of this approach has been shown in a study of mass treatment in Papua New Guinea.^4^ A key principle inherent in an eradication campaign is the need to intervene everywhere the disease occurs. However, the present geographic extent of yaws is incompletely known, because yaws is not a notifiable disease in many affected countries. To guide the WHO eradication programme, a better knowledge of yaws epidemiology is needed.

Data that can be used to identify the burden of yaws in a community include the prevalence of active infectious yaws (ie, ulcers or papilloma), which shows the intensity of yaws transmission, and the prevalence of latent yaws (ie, seropositivity in healthy individuals), which shows the extent of latent or hidden infection in the community. Clinical surveys for active yaws lesions can be done without any sophisticated laboratory test through interviews and physical examinations, whereas serological tests measuring yaws antibody (treponemal and non-treponemal) are needed for surveys of latent disease.^5^ Another important source of information is national routine surveillance data, which allow estimation of the incidence of yaws at country and regional levels; countries report the number of cases at the first administrative level.

In this study, we undertook a systematic review of published and unpublished work to improve our understanding of yaws epidemiology stratified by country, and to provide an update on the number of people with active yaws to estimate at-risk populations in endemic countries.

## Methods

### Search strategy and selection criteria

We did a systematic review to identify all relevant studies that examined yaws prevalence and incidence. We searched PubMed and WHO databases for (“yaws” OR “treponematosis” AND “prevalence” OR “incidence”) OR (“yaws” AND [each individual previous and current yaws-endemic country]^6^). We consulted the Department for the Control of Neglected Tropical Diseases at WHO regarding previous and present yaws-endemic countries.^6^ We limited the search to studies published between Jan 1, 1990, and Dec 31, 2014. This period covers studies published since the last systematic review of yaws epidemiology, which was published in 1992.^7^ No language restrictions were set for searches. We hand-searched the reference lists of all recovered documents for additional references. We also searched for ongoing or recently completed but unpublished studies from the WHO yaws surveillance network.

We included studies if they investigated active or latent yaws prevalence or incidence. Studies on active yaws had to meet the surveillance case definition provided by WHO:^8^ a person with a history of residence in an affected area who presents with signs of clinically active yaws, consisting of chronic skin ulcers, multiple papillomata, squamous macules, bone or joint lesions, or plantar hyperkeratosis. For latent yaws seroprevalence studies, we deemed serological test rapid plasma reagin titres of at least 1:2 and venereal disease research laboratory titres of at least 1:2 as acceptable evidence of untreated latent infection. Use of the treponemal test (*T pallidum* haemagglutination assay, *T pallidum* particle agglutination assay, and the fluorescent treponemal antibody absorption) alone was not sufficient evidence of latent infection because people who have had yaws at any time will test positive for life, even after successful treatment.

### Procedures

We calculated the number of people with active disease at the first administrative level (eg, province, region, and prefecture) between Jan 1, 2010, and Dec 31, 2013. First, whenever possible, we obtained the country estimates of yaws cases at the first administrative level from the latest national reporting figures provided to WHO.^6^ Second, for countries for which no recent data were available, we contacted yaws control programme managers to request official national routine surveillance data. To estimate the maximum population at risk of yaws, we made calculations at the second administrative level (eg, district, department, and regency). We contacted yaws control programme managers to request data on the proportion of second-administrative level regions that reported yaws cases in 2012. We summed the population living in endemic districts using the 2012 reported populations.

### Statistical analysis

For all qualifying studies, we extracted data on study country, sample size, diagnostic test used, number of people with latent or active yaws, and age range. We undertook descriptive analyses of the extracted data. Prevalence estimates are presented for each study with 95% CIs on the basis of binomial distribution. We did not undertake quantitative meta-analyses because the studies we identified did not sample populations at random and hence the estimates are not representative for a broader geographical area. All statistical analyses were done using Stata version 13.1.

### Role of the funding source

There was no funding source for this study. The corresponding author had full access to all the data in the study and had final responsibility for the decision to submit for publication.

## Results

**Figure 1:**
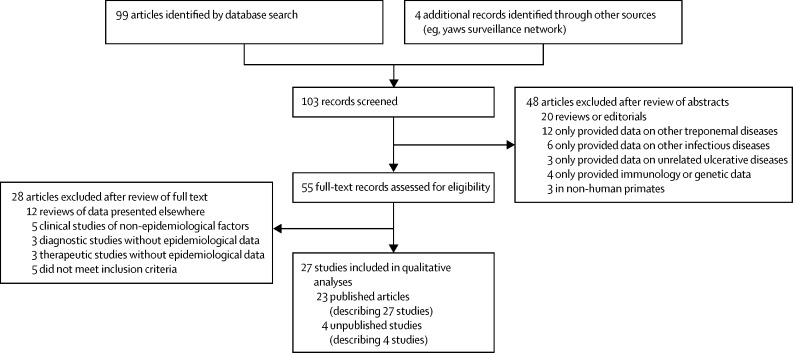
Selection of eligible articles

Our systematic review identified 103 records, from which we identified 23 eligible published articles^9^, ^10^, ^11^, ^12^, ^13^, ^14^, ^15^, ^16^, ^17^, ^18^, ^19^, ^20^, ^21^, ^22^, ^23^, ^24^, ^25^, ^26^, ^27^, ^28^, ^29^, ^30^, ^31^ that described 27 studies that met our inclusion criteria (figure 1). We included data from an additional four studies identified from other sources (personal communications with country managers and yaws experts: Tabah EN, personal communication; Boua B, personal communication; Nsiire A, personal communication; Ayelo G, personal communication). The included studies covered 18 countries. Three of these countries—Guyana, Nigeria, and Wallis and Futuna—were classified by WHO as previously endemic countries with unknown status in 2012. Two countries—Ecuador and India—were reported to have eliminated yaws.^29^, ^31^ The remaining 13 countries were classified as known endemic countries in 2012.^6^

Among the 31 studies, 16 reported data on active yaws prevalence (table 1; Tabah EN, personal communication; Boua B, personal communication; Nsiire A, personal communication).^9^, ^10^, ^13^, ^14^, ^16^, ^18^, ^19^, ^20^, ^21^, ^24^, ^28^, ^29^, ^30^ Patients with suspected yaws skin lesions were further tested with syphilis serology, except in four studies in which diagnosis was made on the basis of clinical criteria only (Tabah EN, personal communication; Boua B, personal communication).^16^, ^19^ After excluding one study from Ecuador^29^ in 1998 in which no clinical cases were detected, prevalence of active yaws lesions ranged from 0·31% in Sumatra, Indonesia,^18^ to 14·54% around the city of Port Moresby, Papua New Guinea.^21^ High prevalence rates were also noted in surveys done in tropical forests in Central Africa that were inhabited by indigenous populations (ie, Pygmies), including 9·03% in Cameroon (Tabah EN, personal communication), 11·34% in the Central African Republic (Boua B, personal communication), 4·77% in the Democratic Republic of the Congo,^14^ and 2·95% in the Republic of Congo.^10^

Overall, eight studies reported data on the prevalence of latent yaws (table 1; Ayelo G, personal communication).^9^, ^25^, ^26^, ^27^, ^28^, ^29^, ^31^ After excluding one study from India^31^ in which no seropositive cases were detected, prevalence of reactive serology ranged from 2·45% in Benin (Ayelo G, personal communication) to 31·05% in Tanna Island, Vanuatu.^26^ Seroprevalence estimates were high in all three studies from the western Pacific region.^25^, ^26^, ^27^ Other studies reporting high seroprevalence were done in Lobaye, Central African Republic (19·72%).^9^ In Ecuador, after the implementation of a yaws surveillance and treatment programme, serological surveys done in 1998 showed a low prevalence of reactive serology (3·54%),^29^ and a survey in India in 2005 reported no sero-reactors among 3821 children younger than 5 years.^31^

Table 2 summarises health-facility-based incidence studies that used passive case finding.^11^, ^12^, ^15^, ^17^, ^21^, ^22^, ^23^ In the study in Nigeria, the results for skin diseases were reported in 2001, mainly in adults, but no cases of yaws were noted.^17^ Among the remaining studies, incidence of yaws ranged between 0·15 cases per 1000 population-years in Côte d'Ivoire^11^ to 25·56 per 1000 population-years in a highly endemic area of Papua New Guinea.^22^

**Figure 2:**
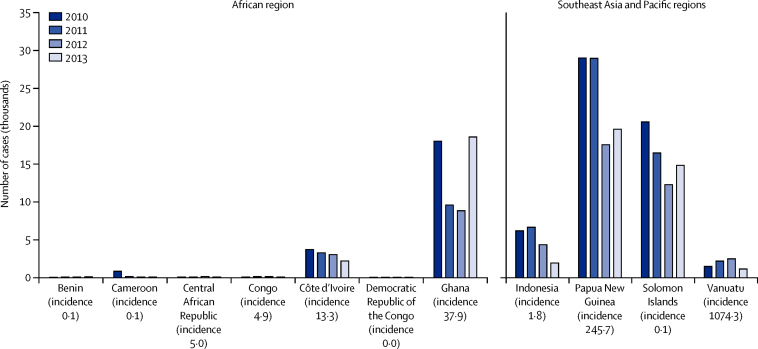
Annual absolute number of yaws cases by country Incidence given in cases per 100 000 population-years in 2010–12.

During the 4-year period between 2010 and 2013, 256 343 yaws cases were reported to WHO from 11 countries and territories (figure 2). Togo and Timor-Leste are judged by WHO to be endemic, but did not report any case in the study period. Large-scale yaws control programmes have recently resulted in disease elimination in two countries (Ecuador and India).^29^, ^31^ Figure 2 shows the annual number of yaws cases in all countries with ongoing transmission. The reported number of active infections was below 300 per year in Benin, Cameroon, Central African Republic, Republic of Congo, and Democratic Republic of the Congo, but data were probably under-reported from all of these countries. 215 308 (84%) of 256 343 cases reported to WHO were from three countries—Papua New Guinea, Solomon Islands, and Ghana.

Table 3 summarises the estimates of the number of people at risk of yaws, stratified by region. We estimated that, in 2012, 8944 8862 people were living in yaws-endemic areas: about 46·7 million people in Africa, 35·8 million in southeast Asia, and 7·0 million in the western Pacific. At-risk population estimates for Ghana, Côte d'Ivoire, and Indonesia might be revised down because not all communities in each endemic district in these countries are endemic for yaws.

**Figure 3:**
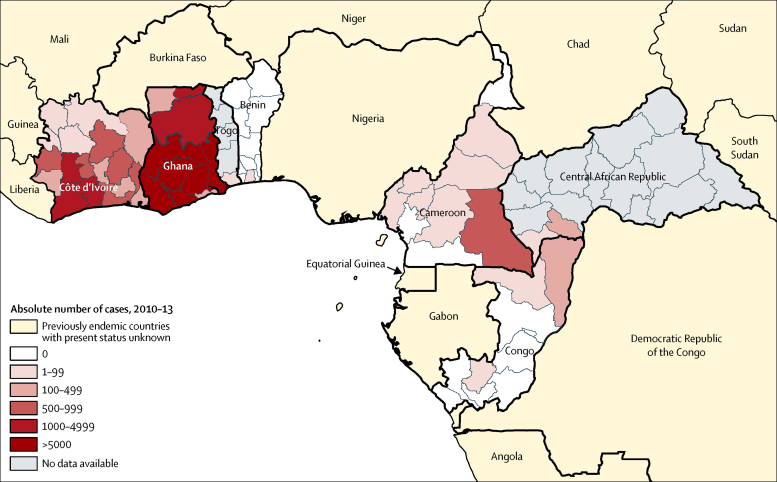
Cumulative number of yaws cases by subnational regions in the WHO Africa region

Figure 3 shows the cumulative number of yaws cases from 2010 to 2013 in the WHO Africa region, shown by subnational regions. Six subnational regions in Ghana were very highly endemic (ie, >5000 cases within the 4-year reporting period), including the Eastern, Central, Volta, Western, Ashanti, and Brong-Ahafo regions. In Côte d'Ivoire, the regions of Fromager, Sud-Bandama, Haut-Sassandra, and Bas-Sassandra were highly endemic (ie, 1000–4999 cases within the 4-year reporting period). The East region in Cameroon, Likouala department in the Republic of Congo, and Lobaye prefecture in Central African Republic, which are close to one another, were all moderately endemic (ie, 100–999 cases within the 4-year reporting period). Data from Central African Republic were limited to surveys in two regions and the situation of the rest of the country remains to be investigated.

**Figure 4:**
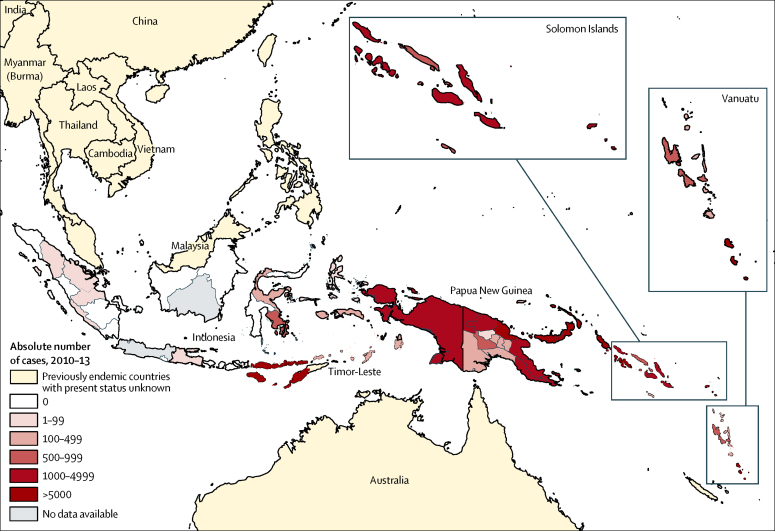
Cumulative number of yaws cases by subnational regions in the WHO southeast Asia and western Pacific regions

Figure 4 shows the cumulative number of yaws cases in the WHO western Pacific and southeast Asia regions within the 4-year period, shown by subnational region. In Papua New Guinea, five provinces were very highly endemic (>5000 cases)—New Ireland, West New Britain, East New Britain, Madang, and Autonomous Region of Bougainville provinces—whereas seven provinces were highly endemic (1000–4999 cases). The Western province in Solomon Islands, and Tafea province in Vanuatu were also very highly endemic. In Indonesia, most cases were found in the province of Nusa Tenggara Timur, where 13 084 cases were reported during the 4-year period. No recent surveillance data have been reported from Timor-Leste, but the country is regarded as endemic according to WHO.

## Discussion

Our data show that about 65 000 yaws cases per year occurred in 13 endemic countries and that in at least 19 countries the incidence of yaws is unknown; thus, there has been limited progress since the last systematic review on yaws epidemiology in 1992 (85 000 yaws cases in 33 endemic countries).^7^ In 1953, Hackett^32^ estimated there were 50–150 million cases of yaws in 90 countries. A substantial decrease in the prevalence of yaws was brought about by the implementation of mass treatment campaigns and subsequent surveillance activities in the 1950s and 1960s. In many countries, yaws control and surveillance activities stopped after 1970, with a subsequent resurgence of yaws, particularly in parts of west and central Africa and in southeast Asia.^7^ Little activity to control the infection has been undertaken since 1990. The scarcity of political will, inadequate funding, and weaknesses in primary health-care systems in affected countries have been the biggest obstacles to the reduction of the burden of yaws in the past two decades.

The methods proposed for assessing yaws burden have not changed substantially since 1953; however, unlike in the previous review by Hackett,^32^ who sent a questionnaire to all countries in Africa and carefully analysed the replies, or in the review by Meheus and Antal,^7^ who compiled original data from country reports submitted to WHO, we also extracted and synthesised a large amount of data from published studies, and complemented this with data from grey literature.

An important finding of our work is that almost 85% of all infections occurred in three countries—Ghana, Papua New Guinea, and Solomon Islands.^6^ The results of individual studies in these countries, which showed high prevalence and incidence rates, are consistent with integrated surveillance data. An overall low number of cases have been reported in national surveillance programmes in other countries in central Africa.^6^ However, we have shown that focal indigenous populations (ie, Pygmies) in the Central African Republic, Cameroon, Republic of Congo, and Democratic Republic of the Congo are affected by yaws, with prevalence of active disease ranging between 3% and 11% (Boua B, personal communication).^9^, ^10^, ^14^ The main risk factor for these groups, as for in other settings in which yaws is highly endemic, is the scarcity of access to health care and poor personal hygiene.

Among the 13 known endemic countries, we estimated that a maximum of about 89 million people were living in yaws-endemic areas. In view of the focal nature of the disease, the size of the population at risk, in particular in Ghana, Côte d'Ivoire, and Indonesia, is uncertain. This global estimate of at-risk individuals would probably be revised down if community-based surveys were used to guide the implementation of mass treatment.

The major limitation of our study is the weakness of routinely reported data. Yaws is not a notifiable disease and the use of national routine surveillance data is likely to result in an underestimation of the real number of cases because yaws predominantly occurs in rural communities with poor access to health facilities, whereas available data are primarily from health facilities. The limited reliability of clinical diagnoses of yaws and the recognition that other organisms can cause clinically similar skin lesions in yaws-endemic countries^33^, ^34^ causes problems for clinical case reporting. The weakness of reported data shows the limitations of the present data and supports the need for surveys as per the WHO strategy.

We did not undertake a meta-analysis for several reasons. First, the studies that we included were primarily implemented in settings where yaws is endemic and no random sampling from a general population was done. Hence, the prevalence estimates are not representative of a given district, province, or an entire country. Second, the number of studies from each WHO region was limited. Third, the inclusion and diagnostic criteria varied markedly between studies, with both children and adults and both clinical and serological definitions of yaws included. These factors make direct comparison of the survey findings difficult.

The results of this systematic review contribute to the epidemiological knowledge needed to guide the preliminary estimation of resources that are necessary for a successful eradication programme. The inability of several countries to undertake more active surveillance and surveys is a major obstacle to achieving the WHO 2020 eradication target. The weaknesses of routinely reported data shows the need to establish a strict and sensitive surveillance system similar to other eradication programmes (eg, for Guinea worm and poliovirus) in a way that enables regionalisation of cases to make the decision about which communities need mass treatment and other control interventions.

**This online publication has been corrected. The corrected version first appeared at thelancet.com on June 8, 2015**

## Figures and Tables

**Table 1:** Characteristics and outcomes of the 24 included studies of active and latent yaws prevalence

		**Year of study**	**Country**	**Location**	**Schoolchildren or community survey**	**Case ascertainment**	**Cases (sample size)**	**Prevalence, % (95% CI)**
**Africa**								
Active yaws assessment								
	Tabah et al (2012; Tabah EN, personal communication)	2012	Cameroon	Lomié, Zoubalot, Messok	Community	Clinical	97 (1075)	9·02 (7·38–10·90)
	Herve et al (1992)^9^	1990	Central African Republic	Lobaye	School children	VDRL and TPHA	12 (213)	5·63 (2·94–9·63)
	Boua et al (2012; Boua B, personal communication)	2012	Central African Republic	Lobaye, Sangha-Mbaeré	School children	Clinical	230 (2030)	11·33 (9·98–12·79)
	Coldiron et al (2013)^10^	2012	Republic of Congo	Bétou, Ebyellé	Community	RDT	183 (6215)	2·94 (2·54–3·40)
	Konan et al (2007)^13^	2004	Côte d'Ivoire	Adzopé	Community	RPR	11 (2182)	0·50 (0·25–0·90)
	Gerstl et al (2009)^14^	2005	Democratic Republic of the Congo	Wasolo	Community	RPR and TPHA	56 (1176)	4·76 (3·62–6·14)
	Nsiire et al (2011; Nsiire A, personal communication)	2011	Ghana	Volta Region	School children	ND	3159 (125 364)	2·52 (2·43–2·61)
	Akogun (1999)^16^	1998	Nigeria	Garkida	Community	Clinical	64 (1523)	4·20 (3·25–5·33)
Latent yaws assessment								
	Ayelo et al (2012; Ayelo G, personal communication)	2012	Benin	Toffo, Zé, Allada	School children	RPR	22 (900)	2·44 (1·54–3·68)
	Herve et al (1992)^9^	1990	Central African Republic	Lobaye	School children	VDRL and TPHA	42 (213)	19·72 (14·60–25·70)
**Western Pacific**								
Active yaws assessment								
	Backhouse et al (1998)^20^	1988	Papua New Guinea	Karkar Island	School children	VDRL, FTA-Abs, and TPHA	26 (632)	4·11 (2·70–5·97)
	Manning and Ogle (2002)^21^	2001	Papua New Guinea	Port Moresby–NCD	School children	VDRL and TPHA	33 (227)	14·54 (10·22–19·81)
	Harris et al (1991)^24^	1989	Vanuatu	Tanna Island	Community	VDRL	464 (20 200)	2·30 (2·09–2·51)
Latent yaws assessment								
	de Noray et al (2003)^25^	2001	Vanuatu	Santo Island	Community	VDRL	57 (273)	20·88 (16·21–26·19)
	Fegan et al (2010)^26^	2008	Vanuatu	Tanna Island	Community	VDRL and TPHA	95 (306)	31·05 (25·90–36·56)
	Guerrier et al (2011)^27^	2010	Wallis and Futuna	Wallis and Futuna	Community	RPR and TPHA	27 (264)	10·23 (6·85–14·53)
**Southeast Asia**								
Active yaws assessment								
	Noordhoek et al (1991)^18^	1988	Indonesia	Sumatra	School children	VDRL, TPHA, FTA-Abs, TmpA EIA, and WB	114 (37 000)	0·31 (0·25–0·37)
	dos Santos et al (2010)^19^	2007	Timor-Leste	Oecusse, Bobonaro, Cova Lima, Atauro Island	Community	Clinical	6 (1535)	0·39 (0·14–0·85)
Latent yaws assessment								
	WHO India (2006)^31^	2005	India	Ten states	School children	RPR and TPHA	0 (3831)	0·00 (0·00–0·00)
**The Americas**								
Active yaws assessment								
	Anselmi et al (1995)^28^	1993	Ecuador	Santiago basin	Community	VDRL and FTA-Abs	16 (1118)	1·43 (0·82–2·31)
	Anselmi et al (2003)^29^	1998	Ecuador	Santiago basin	Community	VDRL and FTA-Abs	0 (1926)	0·00 (0·00–0·19)
	Scolnik et al (2003)^30^	2000	Guyana	Bartica	School children	MHA-TP	52 (1020)	5·10 (3·83–6·63)
Latent yaws assessment								
	Anselmi et al (1995)^28^	1993	Ecuador	Santiago basin	Community	VDRL and FTA-Abs	53 (1118)	4·74 (3·57–6·16)
	Anselmi et al (2003)^29^	1998	Ecuador	Santiago basin	Community	VDRL and FTA-Abs	68 (1926)	3·53 (2·75–4·45)

FTA-Abs=fluorescent treponemal antibody–absorption. MHA-TP=microhaemagglutination assay–*Treponema pallidum*. NCD=National Capital District. ND=not documented. RDT=rapid diagnostic test. RPR=rapid plasma reagin. TmpA EIA=enzyme immunoassay with TmpA antigen. TPHA=*T pallidum* haemagglutination. VDRL=Venereal Disease Research Laboratory. WB=western blot with *T pallidum* subspecies *pallidum* as antigen.

**Table 2:** Characteristics and outcomes of health-facility-based active yaws incidence studies

	**Period of study**	**Country**	**Location**	**Target population**	**Case ascertainment**	**New cases (at-risk population)**	**Incidence, cases per 1000 population-years (95% CI)**
**Africa**							
Toure et al (2007)^11^	2000	Côte d'Ivoire	Nationwide	Children and adults	Clinical	9212 (15 882 758)	0·58 (0·57–0·59)
Konan et al (2013)^12^	2011	Côte d'Ivoire	Nationwide	Children and adults	Clinical	3343 (22 594 212)	0·15 (0·14–0·15)
Edorh et al (1994)^15^	1991	Togo	Nationwide	School children	Clinical	3750 (3 787 000)	0·99 (0·96–1·02)
Nnoruka (2005)^17^	1999–2001	Nigeria	Enugu Hospital	Children and adults	Clinical	0 (2871)	0·00 (0·00–1·28)
**Western Pacific**							
Manning and Ogle (2002)^21^	2000–01	Papua New Guinea	Port Moresby	Children and adults	RPR and TPHA	494 (20 000)	24·70 (22·59–26·95)
Mitja et al (2011)^22^	2009	Papua New Guinea	Lihir Island	School children	RPR and TPHA	138 (5 400)	25·56 (21·51–30·12)
Ministry of Health, Solomon Islands (2013)^23^	2012	Solomon Islands	Nationwide	Children and adults	Clinical	12 372 (515 870)	23·98 (23·57–24·40)

RPR=rapid plasma reagin. TPHA=*Treponema pallidum* haemagglutination.

**Table 3:** Estimates of at-risk populations living in districts judged to be endemic (second administrative level; 2012)

	Population of country*	**Health districts reporting yaws (n/N [%])**	**Population living in endemic districts**
**Africa**			
Benin†	9 364 619	2/34 (5·9%)	Minimum 632 488. Total not known
Cameroon	22 128 420	22/179 (12·3%)	2 360 944
Central African Republic‡	4 600 125	2/17 (11·8%)	Minimum 434 521. Total not known
Republic of Congo	4 001 831	16/84 (19·0%)	Minimum 1 555 513
Côte d'Ivoire	23 261 022	56/81(69·1%)	18 000 000
Democratic Republic of the Congo	75 507 000	ND/36	Not known§
Ghana	24 658 823	160/170 (94·1%)	23 178 000
Togo	6 191 155	2/35 (5·7%)	545 729
**Western Pacific**			
Papua New Guinea	7 146 240	75/89 (84·3%)	6 201 393
Solomon Islands	515 870	10/10 (100%)	515 870
Vanuatu	234 023	6/6 (100%)	234 023
**Southeast Asia**			
Indonesia	241 692 190	106/497(21·3%)	34 588 881
Timor-Leste	120 1500	13/13 (100%)	120 1500

ND=no data.

* From 2012, except Ghana (2010) and Vanuatu and Indonesia (2009).

† Accurate data were only available for two districts. The prevalence of yaws in the remaining 32 districts was not known.

‡ Accurate data were only available for two districts. The prevalence of yaws in the remaining 22 districts was not known.

§ District-level data were not available to allow an accurate calculation of the population at risk.

## References

[1] Mitjà O, Asiedu K, Mabey D (2013). Yaws. Lancet.

[2] WHO (2012). Eradication of yaws—the Morges strategy. Wkly Epidemiol Rec.

[3] Mitjà O, Hays R, Ipai A (2011). Single-dose azithromycin versus benzathine benzylpenicillin for treatment of yaws in children in Papua New Guinea: an open-label, non-inferiority, randomised trial. Lancet.

[4] Mitjà O, Houinei W, Moses P (2015). Mass treatment with single-dose azithromycin for yaws. N Engl J Med.

[5] Hackett CJ, Guthe T (1956). Some important aspects of yaws eradication. Bull World Health Organ.

[6] WHO (2014). Global health observatory data repository: yaws. http://apps.who.int/gho/data/node.main.NTDYAWSEND?lang=en.

[7] Meheus A, Antal GM (1992). The endemic treponematoses: not yet eradicated. World Health Stat Quart.

[8] WHO (2012). Summary report of a consultation on the eradication of yaws. 5–7 March 2012.

[9] Herve V, Kassa Kelembho E, Normand P, Georges A, Mathiot C, Martin P (1992). Resurgence of yaws in Central African Republic. Role of the Pygmy population as a reservoir of the virus. Bull Soc Pathol Exot.

[10] Coldiron M, Obvala D, Mouniaman-Nara I, Pena J, Blondel C, Porten K (2013). The prevalence of yaws among the Aka in the Congo. Med Sante Trop.

[11] Toure B, Koffi NM, Assi KP, Ake O, Konan DJP (2007). Yaws in Côte d'Ivoire: health problem forgotten and neglected. Bull Soc Pathol Exot.

[12] Konan DJP, Aka J, Yao KJ, Kouassi-Gohou V, Yao KE, Faye-Kette H (2013). Update on a neglected tropical disease from the routine health information system in Côte d'Ivoire: yaws, 2001 to 2011. Med Sante Trop.

[13] Konan YE, M'Bea KJ, Coulibaly A (2007). A description of the yaws infection and prevention conditions in the health district of Adzopé. Sante Publique.

[14] Gerstl S, Kiwila G, Dhorda M (2009). Prevalence study of yaws in the Democratic Republic of Congo using the lot quality assurance sampling method. PLoS One.

[15] Edorh AA, Siamevi EK, Adanlete FA (1994). Resurgence of endemic yaws in Togo. Cause and eradication approach. Bull Soc Pathol Exot.

[16] Akogun OB (1999). Yaws and syphilis in the Garkida area of Nigeria. Zentralbl Bakteriol.

[17] Nnoruka EN (2005). Skin diseases in south-east Nigeria: a current perspective. Int J Dermatol.

[18] Noordhoek GT, Engelkens HJ, Judanarso J (1991). Yaws in West Sumatra, Indonesia: clinical manifestations, serological findings and characterisation of new *Treponema* isolates by DNA probes. Eur J Clin Microbiol Infect Dis.

[19] dos Santos MM, Amaral S, Harmen SP, Joseph HM, Fernandes JL, Counahan ML (2010). The prevalence of common skin infections in four districts in Timor-Leste: a cross sectional survey. BMC Infect Dis.

[20] Backhouse JL, Hudson BJ, Hamilton PA, Nesteroff SI (1998). Failure of penicillin treatment of yaws on Karkar Island, Papua New Guinea. Am J Trop Med Hyg.

[21] Manning LA, Ogle GD (2002). Yaws in the periurban settlements of Port Moresby, Papua New Guinea. P N G Med J.

[22] Mitja O, Hays R, Ipai A (2011). Outcome predictors in treatment of yaws. Emerg Infect Dis.

[23] Ministry of Health, Division of Planning and Policy National Health Statistics Office, Solomon Islands (2013). Annual health report 2012, Tech Rep, Solomon Islands, 2013.

[24] Harris M, Nako D, Hopkins T (1991). Yaws infection in Tanna, Vanuatu 1989. Southeast Asian J Trop Med Public Health.

[25] de Noray G, Capuano C, Abel M (2003). Campaign to eradicate yaws on Santo Island, Vanuatu 2001. Med Trop (Mars).

[26] Fegan D, Glennon MJ, Thami Y, Pakoa G (2010). Resurgence of yaws in Tanna, Vanuatu: time for a new approach?. Trop Doct.

[27] Guerrier G, Marcon S, Garnotel L (2011). Yaws in Polynesia's Wallis and Futuna Islands: a seroprevalence survey. N Z Med J.

[28] Anselmi M, Araujo E, Narvaez A, Cooper PJ, Guderian RH (1995). Yaws in Ecuador: impact of control measures on the disease in the Province of Esmeraldas. Genitourin Med.

[29] Anselmi M, Moreire JM, Caicedo C (2003). Community participation eliminates yaws in Ecuador. Trop Med Int Health.

[30] Scolnik D, Aronson L, Lovinsky R (2003). Efficacy of a targeted, oral penicillin-based yaws control program among children living in rural South America. Clin Infect Dis.

[31] National Institute of communicable diseases (India), WHO country office for India (2006). Yaws elimination in India: a step towards eradication. http://whoindia.healthrepository.org/handle/123456789/160.

[32] Hackett CJ (1953). Extent and nature of the yaws problem in Africa. Bull World Health Organ.

[33] Mitja O, Lukehart SA, Pokowas G (2014). Haemophilus ducreyi as a cause of skin ulcers in children from a yaws-endemic area of Papua New Guinea: a prospective cohort study. Lancet Glob Health.

[34] Marks M, Chi K-H, Vahi V (2014). Haemophilus ducreyi associated with skin ulcers among children, Solomon Islands. Emerg Infect Dis.

