# Correction to *Lancet Glob Health* 2015; 3: e324–31

**DOI:** 10.1016/S2214-109X(15)00061-3

**Published:** 2015-06-07

*Mitjà O, Marks M, Konan DJP, et al. Global epidemiology of yaws: a systematic review.* Lancet Glob Health *2015; **3:** e324–31*—In figure 4 of this Article, the island group (known as Santa Cruz Islands) shown in the northern tip of Vanuatu (main map and enlargement) actually belongs to Solomon Islands, namely Temotu Province. Additionally, the first name of Lasse S Vestergaard was spelt incorrectly. These corrections have been made to the online version as of June 8, 2015.

